# Two case reports of adult traumatic inferior hip dislocations following road traffic accidents without complications

**DOI:** 10.1097/MS9.0000000000000639

**Published:** 2023-04-24

**Authors:** Arvin Najafi, Parmida Shahbazi, Danoosh Zargar, Niloofar Gholami, Dorsa Hadavi, Mohammad S. Mirhoseini

**Affiliations:** a Orthopedic Surgery Ward, Shahid Madani Hospital, Alborz University of Medical Sciences, Karaj; b Cardiovascular research center, Alborz University of Medical Sciences, Alborz, Iran; c Radiology Department, Imam Khomeini Hospital Complex, Tehran University of Medical Sciences, Tehran, Iran

**Keywords:** case reports, hip dislocation, hip injuries, traffic accidents

## Abstract

Inferior dislocation of the hip is the rarest type of hip dislocation, mainly occurring from high-energy incidents, mostly from road traffic accidents or sports. It is generally an injury among adults.

**Case presentation:**

A 17-year-old male sustained injuries as a pedestrian, and a 30-year-old male sustained a traffic accident as a motorcycle rider. Both patients complained of severe pain in the hip, decreasing range of motion, and the inability to weight bearing. In both, the affected hip joint was fixed in 90° flexion, abduction, and external rotation, and the leg was slightly shorter than the other limb. An X-ray showed the inferior dislocation of the right hip and left hip, respectively, without any sign of fracture. We reduced both dislocations closely after sedation without any complications during follow-ups.

**Conclusion:**

This injury should be treated as an emergency, and reduction performed as soon as possible, within 6 h. It can be managed usually with closed reduction under general anesthesia. Close follow-up is necessary to prevent its complications, including avascular necrosis, associated fractures, neurovascular compromise, and articular cartilage injuries.

## Introduction and importance

Highlights

- Inferior dislocation of the hip is a rare type of hip dislocation occurring mainly in adults due to high-energy incidents, such as road traffic accidents or sports.
- Two cases of inferior hip dislocation were presented, one in a 17-year-old pedestrian and the other in a 30-year-old motorcycle rider, both successfully treated with closed reduction.
- Early diagnosis and prompt reduction are crucial in managing this injury, and close follow-up is necessary to prevent complications such as avascular necrosis, associated fractures, neurovascular compromise, and articular cartilage injuries.

The frequency of high-energy trauma has increased the incidence of traumatic hip dislocations^1^. The hip joint is inherently stable via the configuration of the bony structure and the strong ligaments that surround it; therefore, a large amount of force is required to dislocate the hip joint^2^.

Hip dislocations can be classified into three primary groups, namely posterior, central, and anterior^3^. Furthermore, anterior dislocations can be subcategorized into superior and inferior dislocations^4,5^.

Inferior dislocation of the hip, also known as luxatio erecta femoris or infra cotyloid dislocation is the rarest type of hip dislocation, with a poorly understood mechanism of injury that mainly occurs due to high-energy incidents, such as road traffic accidents or sports^6^. Traumatic inferior hip dislocation is generally an injury in the adults^2^. Inferior dislocations represent only 2–5% of all hip dislocations, the majority of which are posterior dislocations^6^.

This case study aims to report on the successful close reduction of traumatic inferior hip dislocation in two male patients (aged 17 and 30 years) following road traffic injuries, who did not experience any complications during 2 years follow-ups.

## Case presentation

**Figure 1:**
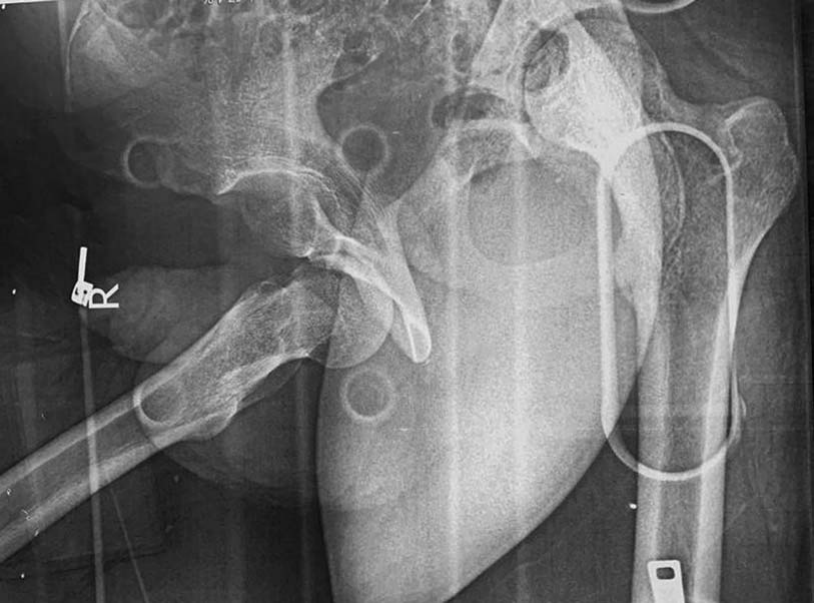
A pelvic obturator Judet view X-ray. An inferior dislocation is seen in the right hip without any sign of fracture.

**Figure 2:**
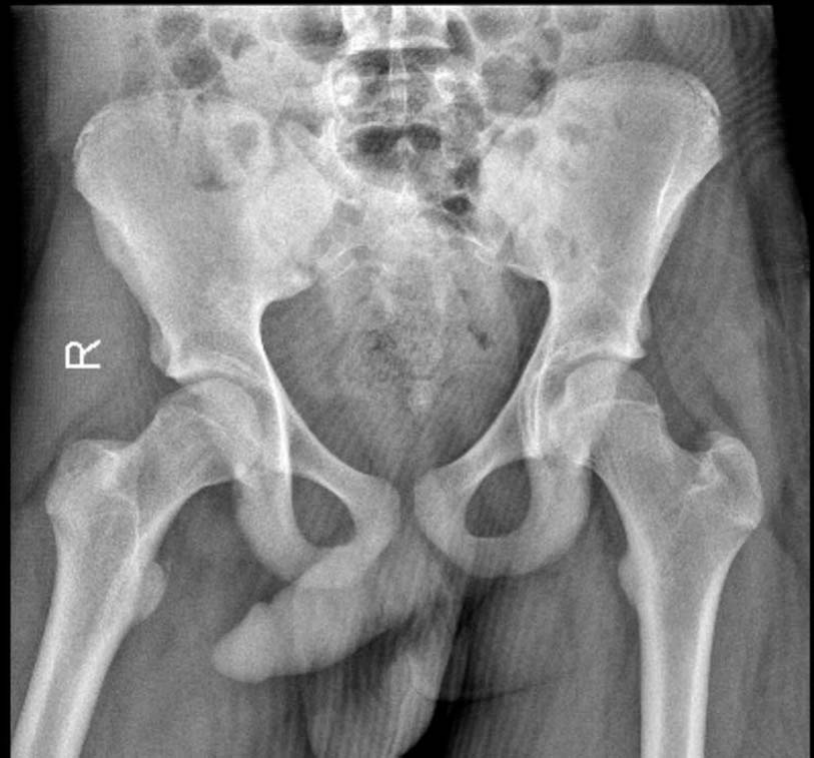
An anterior-posterior pelvic view X-ray after reduction. The femoral head is seen in the acetabulum.

Our first case was a 17-year-old male brought to our emergency department by Emergency Medical Service (EMS) after sustaining injuries in a roadside accident. He was hit by an automobile from the lateral side while crossing the street. He was rolled over by the automobile and could not remember the moment exactly. On arrival, the patient was stable, with a Glasgow coma scale of 15/15. Primary and secondary surveys were performed. He complained of severe pain in his right hip, a decrease in the right hip joint range of motion, and an inability to weight bearing. On the initial examination, severe tenderness was determined in the right hip joint. The right hip joint was fixed in 90° flexion, abduction, and external rotation, and the leg was slightly shorter than the other limb. The straight leg raising test of the right limb was impaired. During the neurovascular examination, the obturator nerve was found to be intact. We checked the neurovascular components, including sciatic nerve branches (tibial nerve and common fibular nerve) and pulse of posterior tibialis and dorsalis pedis arteries, and all were normal. He had no significant past medical history or drug history. After achieving vascular access and analgesic administration, the patient was redirected to radiological imaging. An obturator Judet view X-ray was taken (Fig. 1). An inferior right hip dislocation was found without any sign of fracture. We planned to reduce the dislocation closely. Thus, we transferred the patient to the operating room, and after sedation, the reduction was done with the following maneuvers: 1 – Counter-traction using a sheet passed under his hip, 2 – Adduction, 3 – Internal rotation, 4 – Traction, 5 – Abduction, and 6 – External rotation. After reduction, the neurovascular examination remained normal. To confirm the reduction, an anterior-posterior (AP) pelvic view X-ray was taken (Fig. 2). The reduction was successful and there were no signs of fracture in the imaging. The patient underwent immobilization for a period of 6 weeks and was monitored for any pain, limping, or complications related to osteonecrosis for a duration of 2 years.

**Figure 3:**
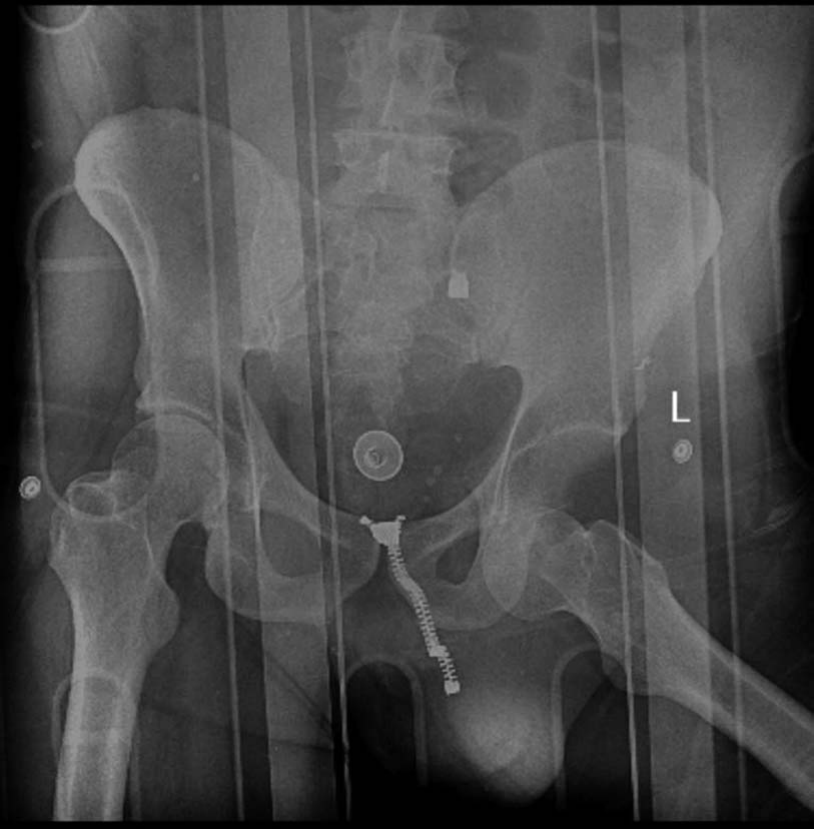
An anterior-posterior pelvic view X-ray. An inferior hip dislocation is seen in the left hip. No sign of fracture is seen.

**Figure 4:**
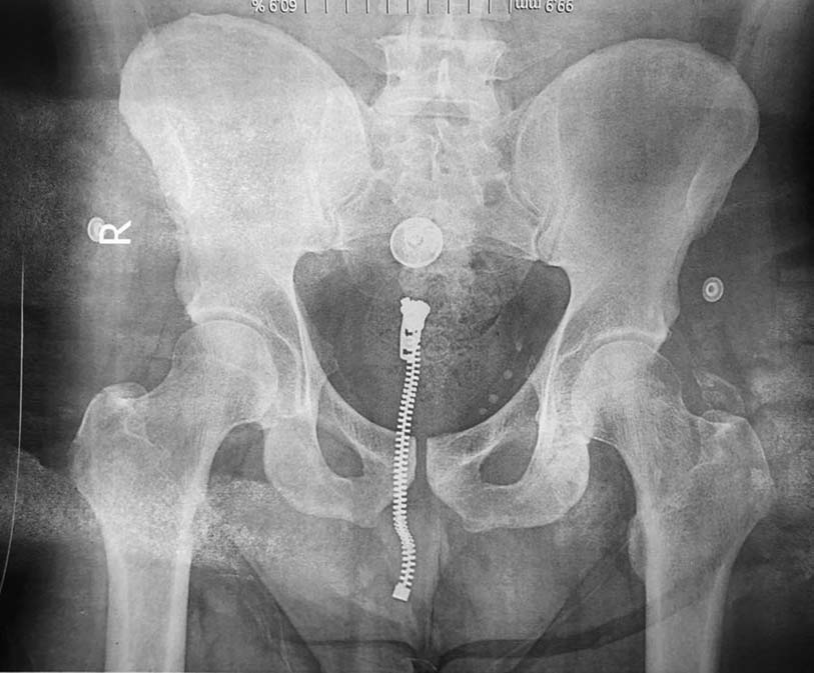
A post-reduction anterior-posterior pelvic view X-ray. The femoral head is seen in the acetabulum.

Our second case was a 30-year-old male who sustained a traffic accident as a motorcycle rider, brought to our emergency department by EMS. He was riding a motorcycle with a speed of 60 kmph, while being crushed by an automobile from the lateral side. He was thrown from the motorcycle but could not remember the scene of the accident. On admission, the patient had stable vital signs. The Glasgow coma scale was 15/15. Primary and secondary surveys were performed. His chief complaint was severe pain in his left hip. He could not move his left hip joint properly and was unable to bear his weight. On physical examination, severe tenderness was notable in the left hip joint. The left hip joint was fixed in 90° flexion, abduction, and external rotation, and the leg appeared shorter than the other limb. The straight leg raising test was not normal on the left lower limb. Evaluation of the neurovascular components, including sciatic nerve branches (tibial nerve and common fibular nerve), obturator nerve, and pulse of posterior tibialis and dorsalis pedis arteries, was performed without any abnormal findings. The patient’s medical and drug history was insignificant. Intravenous (i.v.) administration of analgesic was ordered, and the patient underwent radiological imaging. An AP pelvic view X-ray was taken (Fig. 3). An inferior left hip dislocation was diagnosed without any fractures. The close reduction was planned as the treatment of choice. Therefore, the patient was sedated in the operating room and the reduction was performed with the following maneuver: 1 – Counter-traction using a sheet passed under his hip, 2 – Adduction, 3 – Internal rotation, 4 – Traction, 5 – Abduction, and 6 – External rotation. After reduction, the neurovascular remained intact. An AP pelvic X-ray was taken (Fig. 4) as follow-up imaging for confirmation of the reduction. The reduction was successful without a sign of fracture in the imaging. The patient was immobilized for 6 weeks and was followed up for 2 years for pain, limping, or any osteonecrosis complications.

The entire procedure, including reduction and follow-up care, was performed by the first author, who is an orthopedic surgeon with 10 years of experience.

This paper has been reported in line with the SCARE (Surgical CAse REport) criteria^7^.

## Clinical discussion

Hip dislocation often occurs with high-energy trauma since the hip joint is stabilized by surrounding thick, strong muscles and ligaments^2^. The literature revealed 16 studies reporting 34 cases related to luxatio erecta, inferior dislocation, or infra cotyloid dislocation of the hip, the majority of which being in non-English literature. Until 2006, only five cases were reported among adults^8^. Dislocation could result from a high fall, leading to an axial load to the femur in a flexed position, or from an injury that forces the hip into wide abduction^2^. The exact mechanism leading to the deformity is not always remembered and has not been described in detail because the reported cases were children or teenagers^8^. There are two types of inferior hip dislocation: the ischial type and the obturator type. The ischial type is more common than the obturator type. In the obturator type, when a force is applied to the femur while the hip joint is in abduction and flexion, and external rotation, the femoral head dislocates anteriorly and inferiorly to the obturator foramen^1,2^. In the ischial type, a force is applied to the femur, while the hip and knee joints are in hyperflexion without abduction or external rotation^2^. The patient presents with pain and usually supports an acutely flexed thigh with his or her hands, which rests against the lower abdomen parallel, or nearly so, with the long axis of the body^1^. The femoral head dislocates inferiorly next to the ischium^2^. Inferior hip dislocation can be managed usually with closed reduction under general anesthesia and sufficient sedation. Reduction is usually achieved by maintaining traction and gradual knee extension with some internal rotation if needed^6^. Concomitant intertrochanteric and femoral neck fractures could also be seen, and these fractures should be treated after the reduction of the hip joint. Post-reduction recommendations include 1–6 weeks of immobilization followed by partial weight bearing^8^. With all types of hip dislocations, there is always a risk for complications which could include avascular necrosis (AVN) in around 10%, associated fractures in less than 40%, neurovascular compromise in less than 25%, and articular cartilage injury in about 6% of the cases. Therefore, this kind of injury should be treated as an emergency, and reduction should be performed as soon as possible within 6 h of surgery under sufficient anesthesia and muscle relaxant to reduce the chances of AVN^6^. Post-treatment rehabilitation does not influence avascular necrosis or re-dislocation in inferior hip dislocation because the incidence of AVN is related to the interval from injury to reduction rather than time to weight bearing^1^.

## Conclusion

Inferior hip dislocation is a rare type of hip dislocation associated with high-energy trauma, but it may lead to a life-threatening condition. Diagnosis of this injury is evident clinically based on the position of the limb at presentation and lately through radiological imaging. Usually, this type of dislocation is treated with closed reduction. Follow-up is important to monitor any signs of AVN.

## Ethical approval

Our study is a case report without any intervention on the patient, and informed consent has been obtained for publication.

## Consent

Written informed consent was obtained from the patients for the publication of this case report and accompanying images. A copy of the written consent is available for review by the Editor-in-Chief of this journal on request.

## Sources of funding

None.

## Author contribution

A.N.: idea, supervision, revising the manuscript, confirming final draft, and guaranteeing all details of the project; P.S. and D.Z.: data collection, literature review, writing the initial draft, revising the manuscript, confirming final draft, and guaranteeing all details of the project; N.G. and D.H.: data collection, revising the manuscript, confirming final draft, and guaranteeing all details of the project; M.S.M.: supervision, revising the manuscript, confirming final draft, and guaranteeing all details of the project.

## Conflicts of interest disclosure

The authors declare no conflicts of interest.

## Guarantor

Arvin Najafi, Parmida Shahbazi, Danoosh Zargar, Niloofar Gholami, Dorsa Hadavi, and Mohammad Sajad Mirhoseini.

## Provenance and peer review

Not commissioned, externally peer-reviewed.

## References

[1] Singh R Sharma SC Goel T . Traumatic inferior hip dislocation in an adult with ipsilateral trochanteric fracture. J Orthop Trauma 2006;20:220–222. 16648705 10.1097/00005131-200603000-00010

[2] Tekin AÇ Çabuk H Büyükkurt CD . Inferior hip dislocation after falling from height: a case report. Int J Surg Case Rep 2016;22:62–65. 27058153 10.1016/j.ijscr.2016.02.041 PMC4832043

[3] Yeganeh A Tavakoli N Soleimani M . Inferior hip dislocation in a 60-year-old man; a case report. Arch Acad Emerg Med 2022;10:e17. 35402998 10.22037/aaem.v10i1.1498 PMC8986494

[4] Day MA Duchman KR Noiseux NO . Traumatic obturator dislocation following total hip arthroplasty managed with closed reduction: a case report and review of the literature. JBJS Case Connect 2017;7:e66. 29356706 10.2106/JBJS.CC.17.00042

[5] Syam K Saibaba B Aggarwal S . Update review and clinical presentation in adult inferior dislocation of hip. Eur J Orthop Surg Traumatol 2017;27:1039–1044. 28210820 10.1007/s00590-017-1918-8

[6] Ismael S Vora J Thomas P . Adult traumatic inferior hip dislocation: rare case ended with open reduction. J Orthop Case Rep 2017;7:101. 28630852 10.13107/jocr.2250-0685.708 PMC5458685

[7] Agha RA Franchi T Sohrabi C . for the SCARE Group. The SCARE 2020 guideline: updating consensus Surgical CAse REport (SCARE) guidelines. Int J Surg 2020;84:226–230. 33181358 10.1016/j.ijsu.2020.10.034

[8] El Hajj Moussa M Tawk C Hoyek F . Traumatic inferior hip dislocation: a rare adult case with ipsilateral bifocal hip fracture. J Surg Case Rep 2016;2016:rjw056. 27141043 10.1093/jscr/rjw056 PMC4852974

